# *Yersinia pestis* Plasminogen Activator Gene Homolog in Rat Tissues

**DOI:** 10.3201/eid1902.120659

**Published:** 2013-02

**Authors:** Ingmar Janse, Raditijo A. Hamidjaja, Chantal Reusken

**Affiliations:** Author affiliation: National Institute for Public Health and the Environment, Bilthoven, the Netherlands

**Keywords:** Yersinia pestis, bacteria, plasmimogen activator, false positive value, pla, rats, rodents, gene homolog, gene homologue, zoonoses, plague, antibacterial, antimicrobial, *Enterobacteriaceae*

**To the Editor***: Yersinia pestis* causes plague, which primarily affects rodents, but is an invasive and virulent pathogen among humans. *Y. pestis* infection is endemic in small rodent populations in different parts of the world, and the bacterium is considered a potential bioweapon because it can be easily isolated, produced, dried, and dispersed as an aerosol. Antimicrobial drug treatment can be lifesaving during the early stages of illness; hence, rapid and sensitive methods for *Y. pestis* detection in environmental and clinical samples are required. Multiple PCR assays for *Y. pestis* detection that primarily detect markers located on plasmids have been developed (*1*–*6*). The plasminogen activator/coagulase (*pla*) gene, located on plasmid pPCP1, is incorporated into most *Y. pestis* PCR assays, and in several studies it was the prime or sole marker (*1*,*2*,*5*,*7*–*9*). Reasons for including *pla* in these assays are its occurrence in multiple copies, its absence from closely related *Yersinia* species, and its role in *Y. pestis* virulence (*1*,*4*,*5*).

While validating the specificity of a multiplex qPCR assay for the detection of *Y. pestis* (*6*), we obtained DNA from the dissected peritoneum of a black laboratory rat (*Rattus rattus*), which tested positive for the *pla* gene. Two other *Y. pestis* signature sequences were not amplified. Additional samples were analyzed from black (n = 11) and brown (*Rattus norvegicus* [n = 4]) rats that had been caught on poultry and pig farms in the southeastern region of the Netherlands during 2008. Positive indicators for *pla* were found in samples from 8 of these black rats and in samples from 2 of the brown rats. Samples from 2 laboratory rats tested negative for *pla*. Inferences of the incidence of *pla*-positive rats cannot be made because of low sample numbers and potential bias in capturing rats that had putative infections. 

To exclude the possibility of contamination of host DNA with DNA from intestinal flora during isolation of the peritoneum, we examined the occurrence of *pla* in other tissues. Lung and liver samples were available from all 17 rats, and leg tissue samples were available from 7 rats, 5 of which had positive peritoneal tissue test results. The leg and lung tissues of 1 rat and the leg tissue of another rat tested positive, albeit at considerably lower quantities (higher quantification cycles) than *pla* values measured in peritoneal samples. These results did not support the likelihood of contamination during sampling or the occurrence of local infections; they did support the hypothesis of a systemic infection in the rats. To investigate whether the presence of the *pla* gene sequences indicated the presence of the carrier pPCP1 plasmid in *Y. pestis*, we designed PCR assays for the amplification of 3 conserved regions of this plasmid (Technical Appendix). Each assay produced PCR products from *Y. pestis*; only the transposase gene was amplified from *Y. pseudotuberculosis*. None of the PCR assays amplified DNA from samples collected from rats.

*Pla* genes obtained from 2 of the peritoneum samples collected from black rats were sequenced and appeared to be identical (GenBank accession no. HQ606074). Alignment with *Y. pestis pla* genes, which are highly conserved among *Y. pestis* isolates, revealed 11 nt differences in 880 bp (98.8% similarity). A BLAST (http://blast.ncbi.nlm.nih.gov/Blast.cgi) search retrieved such highly similar genes only from *Y. pestis* sequences; the next most similar sequences of other *Enterobacteriaceae* were at 78% similarity.

The genewalking PCR procedure was used to explore sequences adjacent to the *pla* gene (Technical Appendix). One PCR product was sequenced and appeared to be in part homologous to the *pla* gene, but the adjacent sequence displayed high homology to genes coding for replicon (*rep)* proteins in several bacterial genera in the family *Enterobacteriaceae*, e.g., *Escherichia*, *Shigella*, and *Salmonella*. The existence of a concatenated *pla-rep* sequence in rat tissue samples was confirmed by amplification of a PCR product from a primer targeting the *pla* gene, combined with a primer targeting the *rep* gene sequence that was acquired by using the genewalking procedure. The resulting 223-bp PCR product (GenBank accession no. JQ756394) consisted of a 141-bp sequence identical to the *Y. pestis pla* gene, linked to a 72-bp sequence that was 97% similar to enterobacterial *rep* protein genes. Attempts to obtain more sequence information from *rep* sequences by using primers derived from conserved domains in enterobacterial *rep* genes were unsuccessful. This suggests that the *pla-rep* sequence is derived from uncharacterized bacteria. *Rep* proteins function as replication activators of their carrier plasmids. 

The *rep* sequences identified in this study were most similar to those of plasmids involved in bactericidal activity, a function that is also ascribed to the bacteriocin pesticin gene clusters of *Y. pestis* pPCP1 plasmids. The occurrence in unknown organisms that have *pla* genes that are similar to *Y. pestis pla* genes has consequences for the detection of *Y. pestis*. To prevent false positive results*,* detection protocols should include at least 1 supplemental target to confirm the presence of *Y. pestis* (*6*). In addition, investigators using *pla* gene analysis, for instance, while reconstructing ancient plague epidemics (*10*), should be aware of the occurrence of these homologs.

Technical Appendix Primers used in *Yersinia pestis* plasminogen activator gene homolog in rat tissues.
