# Supplementary material for: Yersinia pestis Plasminogen Activator Gene Homolog in Rat Tissues
Source: Emerg Infect Dis. 2013 Feb;19(2):342–4. doi: 10.3201/eid1902.120659 (PMC3559043; doi:10.3201/eid1902.120659)
Supplement: Technical Appendix — Primers used in Yersinia pestis plasminogen activator gene homolog in rat tissues. [file 12-0659-Techapp-s1.pdf]

Article DOI: http://dx.doi.org/10.3201/eid1902.120659

Yersinia pestis Plasminogen Activator Gene Homolog in Rat Tissue

Technical Appendix

Primers Used in *Yersinia pestis* Plasminogen Activator Gene Homolog in Rat Tissues

Technical Appendix Table 1. Primers for amplification and sequencing of the plasminogen activator gene*

Forward primer	Sequence (5′→3′)	Reverse primer	Sequence (5′→3′)	Position†
*pla*sq-f	ATGAAAATCAATCTGAGTGGACAGATCAC	*pla*sq-r	CAGAAGCGATATTGCAGAC	6990–7602
*pla*sq2-f	TAACTATTCTGTCCGGGAGT	*pla*sq2-r	ATTATCATGTGCCCGAAC	6696–7339

*pla, plasminogen activator/coagulase.
†Position in entry AF053945 from *Y. pestis* strain KIM5.

Technical Appendix Table 2. Primers for amplification and detection of conserved regions on plasmid pPCP1*

Forward primer	Sequence (5’→3’)	Reverse primer	Sequence (5’→3’)	Position†	Present in
pPratTr21-f	TCTCGGTTCCTCAGGAG	pPratTr21-r	TGCACGTCATACTCTTTTTTCT	397–1021	Ype + Yps
pPratPst-f	GAGATGGAGAAAGACAGTGA	pPratPst-r	ATTTTAACAATCCACTATCGATA	4817–5699	Ype
pPratR1-f	AGGCCATGAACGACT	pPratR1-r	GATGGGAAATACAACTACGAAAATTA	2100–2826	Ype

*Ype, *Yersinia pestis*; Yps, *Yersinia pseudotuberculosis.*
†Position in entry AF053945 from *Y. pestis* strain KIM5.

Technical Appendix Table 3. Primers for genewalking

Primer	Sequence (5’→3’)	Function
usPuTSP1	ATTTGGTATTAACTGAGATGAT	Upstream *Pla* TSP1 primer
us*Pla*TSP2	CTGCATTAGCACTCCCGGA	Upstream *Pla* TSP2 primer
us*Pla*TSP3	CCCGGACAGAATAGTTATAATGG	Upstream *Pla* TSP3 primer
ds*Pla*TSP1	CCATTGATAAGAATAGTGGAGA	Downstream *Pla* TSP1 primer
ds*Pla*TSP2	TCTGTCTCTATTGGCGGAGA	Downstream *Pla* TSP2 primer
ds*Pla*TSP3	GCTGCCGGTATTTCCAATAA	Downstream *Pla* TSP3 primer

*Following the instructions of the DNA Walking SpeedUp Premix Kit (Seegene Inc.), gene-specific nested primers targeting the flanking regions of the pla gene were designed. These were used in combination with supplied primers for 3 consecutive rounds of PCR amplification. *Pla*, plasminogen activator/coagulase.

Technical Appendix Table 4. Primers for amplification of the concatenated *pla-rep* sequence*

Forward primer	Sequence (5’→3’)	Reverse primer	Sequence (5’→3’)	PCR product
us*Pla*TSP1	ATTTGGTATTAACTGAGATGAT	*Pla*Rep1	GGTAAATTTTCGTCGAAGTAT	Used for sequencing *pla*-rep
us*Pla*TSP1	ATTTGGTATTAACTGAGATGAT	*Pla*Rep2	TCATCCGTGTGATAGGGAC	Not formed
us*Pla*TSP1	ATTTGGTATTAACTGAGATGAT	*Pla*Rep3	GTCGCTCCAGTCAATCG	Not formed
us*Pla*TSP1	ATTTGGTATTAACTGAGATGAT	*Pla*Rep4	GGCGCGGTTCTCACGG	Not formed
us*Pla*TSP1	ATTTGGTATTAACTGAGATGAT	*Pla*Rep5	ATCGCCCTGTAAGCCCATTT	Not formed
us*Pla*TSP1	ATTTGGTATTAACTGAGATGAT	*Pla*Rep6	TCYTGCCAATAGCCC	Not formed

*Primers *Pla*Rep 2–6 were based on an alignment of conserved domains in enterobacterial *rep* genes. *Pla*, plasminogen activator/coagulase; rep, replicon.
